# Differentiating language-specific from language-related network dynamics using dynamic functional connectivity analysis

**DOI:** 10.1007/s11571-026-10579-0

**Published:** 2026-11-10

**Authors:** Atsuko Kurosu, Jinqing Liang, Ninet Sinaii, Divesh Thaploo, Adebiyi Sobitan, Kristen Wingert, Sunder Rajan, Nadia Biassou

**Affiliations:** 1 The Integrative Neuroscience of Communication Unit, National Institute on Deafness and Other Communication Disorders, Bethesda, MD 20892 USA; 2 Biostatistics and Clinical Epidemiology Service, National Institutes of Health Clinical Center, Bethesda, MD 20892 USA; 3 Department of Radiology and Imaging Sciences, National Institutes of Health Clinical Center, Bethesda, MD 20892 USA

**Keywords:** Functional connectivity, Neuroimaging, Magnetic resonance imaging, Language processing

## Abstract

Language processing is a highly complex dynamic neural activity that requires the recruitment of both regions critical for language processing and large-scale cognitive networks. Because language networks are interconnected and dynamically reconfigure their connections depending on task demand and complexity, it is challenging to differentiate language-specific networks form language-related cognitive networks during both resting-state fMRI (rs-fMRI) and task-based fMRI (tb-fMRI). Prior evidence demonstrated state-dependent differences in dynamic functional connectivity (FC) within identical networks across rest- and task-conditions. Based on this evidence, we hypothesized that examining dynamic FC patterns could help differentiate the functional specialization of large-scale neural networks in the human brain. Thus, we examined whether temporal patterns of dynamic FC could functionally differentiate networks engaged during a language task. Using a data-driven dynamic FC framework, we tested whether rest–task state transitions preferentially modulate subsets of large-scale networks. Specifically, we examined whether monotonic temporal trends in dynamic FC, used as a first-order approximation of sustained task engagement, were more prominent in networks showing task-sensitive dynamics, while others exhibited resting-state-like dynamics. We analyzed rs-fMRI and tb-fMRI data from 172 healthy participants (86 females, 27.6 ± 3.8 years old) from the Human Connectome Project. Dynamic FC analyses revealed significant state-dependent differences in correlation variability between rs-fMRI and tb-fMRI (Bonferroni-corrected *p* < 0.001). A subset of networks exhibited task-sensitive temporal modulation, whereas other networks showed dynamic patterns closely paralleled resting-like dynamics without reaching statistical significance. These findings suggest that dynamic FC provides a useful framework for functionally distinguishing language-specific networks from language-related cognitive networks.

**Supplementary Information:**

The online version contains supplementary material available at https://doi.org/10.1007/s11571-026-10579-0.

## Introduction

Language processing is a highly complex dynamic neural activity that requires the recruitment of large-scale networks, including regions critical for language processing (e.g., left hemisphere frontal and temporal areas) (I. A. Blank and Fedorenko 2017; Fedorenko et al. 2024a; Mineroff et al. 2018; Williams et al. 2022; Wylie and Regner 2014). Large-scale language networks include language-specific networks and language-related cognitive networks (e.g., domain-general), and some networks may overlap (Fedorenko, 2014; Fedorenko et al. 2024a; Geranmayeh et al. 2014; Mineroff et al. 2018; Wylie and Regner 2014). Language-specific networks are involved in phonological, lexico-semantic, syntactic, and pragmatic analyses (Campbell and Tyler 2018). Along with language-specific networks, language-related cognitive networks are coactivated when additional cognitive resources (e.g., attention and working memory) are required for language processing such as when listening in noise and/or processing ambiguous sentences (I. A. Blank and Fedorenko 2017; Campbell and Tyler 2018; Diachek et al. 2020; Fedorenko, 2014). Although both language-specific networks and larger-scale functional brain networks involved in several cognitive acts [e.g., default mode network (DMN)] are involved in semantic processing, there is a theoretical functional dissociation between the language-specific network and the language-related cognitive network (Mineroff, Blank, & Fedorenko, 2018). Yet, language networks are interconnected and are continuously changing their connections depending on task demand and complexity at different timescales (Fedorenko, 2014; Fedorenko and Thompson-Schill 2014; Jung and Lambon Ralph 2023). Hence, for practical purposes, it is difficult to differentiate the kinds of information a language network may be processing at any given moment. For this reason, this study investigated a method to differentiate these two information processing entities in real time across whole brain networks.

There has been increased interest in identifying functional connectivity (FC) during language processing (Biswal and Uddin 2025; Chang and Lambon Ralph 2020; Zhu et al. 2023). FC measures spatial and temporal correlations in blood-oxygen-level dependent (BOLD) signal changes across different brain regions, and there are two types of FC (resting-state FC vs. task-based FC) with two analytical approaches (static vs. dynamic) (Friston et al. 1994; Rosch and Mostofsky 2019). Resting-state fMRI (rs-fMRI) examines spontaneous BOLD MRI signal changes without performing any specific tasks, while task-based fMRI (tb-fMRI) examines brain activity changes during periods that alternately include specific tasks (Klingbeil et al. 2019; Rogers et al. 2007). The static analytical approach does not consider FC temporal fluctuation and measures average time courses of FC while the dynamic approach examines temporal fluctuation of FC over the examination (Calhoun et al. 2014; Cohen 2018). Dynamic functional connectivity can be characterized by multiple temporal properties. Variability metrics (e.g., variance or standard deviation of FC across time window) quantify the dispersion of connectivity strength, whereas temporal trend analyses assess systematic monotonic changes in FC over time. These measures capture distinct aspects of network dynamics and should not be conflated.

A recent study from our lab indicated distinct dynamic FC linear trend over time pattern differences between resting-state and task-based conditions of auditory language comprehension within identical networks in healthy participants (Kurosu et al. 2026). Specifically, we found increased variability in linear correlations over time in specific FC networks during dynamic tb-fMRI, but not in dynamic rs-fMRI in the same participants. Given this evidence, we speculated that examining pattern differences in dynamic FC linear trend over time between rs-fMRI and tb-fMRI can differentiate specific kinds of information processed by a language network (e.g., language-specific vs. language-related). Notably, we distinguish between “language-specific” and “language-related” networks at a functional level rather than an anatomical structure, consistent with previous evidence of a theoretical functional dissociation between these networks (Mineroff, Blank, & Fedorenko, 2018). We hypothesized that dynamic FC during a language task would show greater temporal modulation than resting-state dynamics, and that this modulation would vary across large-scale networks (Duncan 2010; Fedorenko et al. 2011). Specifically, we predicted that networks exhibiting task-sensitive temporal trends in dynamic FC would reflect functional specialization for language processing, whereas networks whose dynamic patterns during tb-fMRI closely resembled resting-state dynamics would be more consistent with language-related, domain-general cognitive processing (e.g., attention, salience, and default mode systems). Importantly, this distinction was not imposed a-priori but rather evaluated based on emergent state-dependent differences in network dynamics. To test these hypotheses, we first examined whether dynamic FC correlations within the same networks differ systematically between rs-fMRI and tb-fMRI, extending our prior findings of state-dependent network dynamics. We then evaluated whether temporal patterns of dynamic FC could functionally distinguish networks preferentially modulated by language processing from those retaining resting-state–like dynamics.

## Materials and methods

### Participants

We conducted respective analyses of both rs-fMRI and tb-fMRI data from the Human Connectome Project (Van Essen et al. 2013). We used Cohort 3 from the HCP S1200 release, comprising 262 age- and sex-matched 262 participants.

The 3T fMRI data from 172 healthy participants (86 males and 86 females, 27.6 ± 3.8 years old) (Table 1) were analyzed for the purposes of the current study. They were selected based on the.

**Table 1:** Demographic information of the participants (*n* = 172)

Variable		
Age (years)	Mean ± SD	27.6 ± 3.8
	Range	22–37
Sex	Male	86 (50%)
	Female	86 (50%)
Handedness	Left	19 (11%)
	Right	151 (88%)
	Unknown	2 (1%)
Education (years)	< 8	0
	9–12	4 (2%)
	12–15	71 (42%)
	16	76 (44%)
	> 16	20 (12%)
	Unknown	1 (< 1%)

several parameters which are: (1) availability of complete language task fMRI data acquired in its entirety, (2) availability of corresponding resting-state fMRI data, and (3) acceptable data quality and head motion across both task and resting-state scans, as defined by pre-defined motion and preprocessing quality control thresholds (Liang et al. 2025). Participants were excluded if task runs were incomplete, resting-state data were missing, or motion/artifact criteria were not met in either condition. Applying these criteria resulted in a final analytic sample of 172 participants with complete and high-quality task and resting-state fMRI data suitable for dynamic functional connectivity analyses. We have explicitly detailed the inclusion and exclusion criteria in the Methods Sect. 88% of the participants were right-handed. The Institutional Review Board approval was obtained at the respective institutions where all participants provided written informed consent. The datasets were made available via permission to the PI from HCP- Washington University St. Louis (Van Essen et al. 2013).

### Experimental design

#### Data acquisition

The S1200 dataset that consists of 3T MRI data including rs- and tb-fMRI data from the Human Connectome Project (HCP) were analyzed. Imaging acquisition was performed using gradient-echo EPI on a Siemens 3T MRI using a standard 32-channel head coil. Imaging parameters were flip angle: 52°, TR: 720ms, TE: 33.1, Voxel size: 2 mm x 2 mm x 2 mm, FOV: 208 × 180 mm; and slice thickness of 2 mm (isotropic voxels), and multiband factor of 8. During rs-fMRI, participants were instructed to lie down still and look at a cross-hair on a dark background for the scan duration. During tb-fMRI, participants completed seven tasks, which were working memory, motor, gambling, social cognition, relational and emotion processing, and language tasks. The current study focused on the language task and math task (control) from the fMRI data. The details of the imaging protocol have been described elsewhere (Van Essen et al. 2013).

Task and acoustic stimuli: The story and math tasks (2 runs; each contains 4 blocks of a story task and 4 blocks of the math task) originally were developed for the Binder et al. (2011) study. The acoustic stimuli from the original study were used during tb-fMRI (Binder et al. 2011). The math task served as a control condition. The acoustic stimuli for both tasks were generated by using a text-to-speech synthesizer, MacinTalk, residing in Mac OS X 10.5. The acoustic stimuli were presented via E-prime software during tb-fMRI.

During the story blocks, participants listened to short stories (mean duration = 29.8 ± 14.5 s; mean number of sentences = 7.0 ± 3.0) adapted from the Aesop’s fables collection and were asked 2-alternative forced choice questions which asked about the topic of each story. Participants answered the questions by pressing a button which was under their right index finger or right middle finger. During the math blocks, participants were asked to calculate serial addition and subtraction. The math tasks also were presented auditorily followed by 2-alternative forced-choice questions. Participants pressed a button to select answers. The phoneme and word rate and speaking style of the acoustic stimuli for the math task were matched to those for the story tasks. The details of the tasks were described in the Binder et al. (2011) study.

The story and math tasks rely both on common and task-specific neuronal resources (Arsalidou and Taylor 2011; Baldo and Dronkers 2007; Kulasingham et al. 2021). Both tasks require verbal working memory, acoustic, phonological, and simple lexical-syntactic processing. In addition to these processes, the math task requires arithmetic concepts while the story task requires more complex syntactic and semantic processing (Binder et al. 2011). Thus, Binder and colleagues (2011) suggested that the neural activation difference between the two tasks represents syntactic and semantic processing during the story task.

#### Data processing

**Fig. 1:**
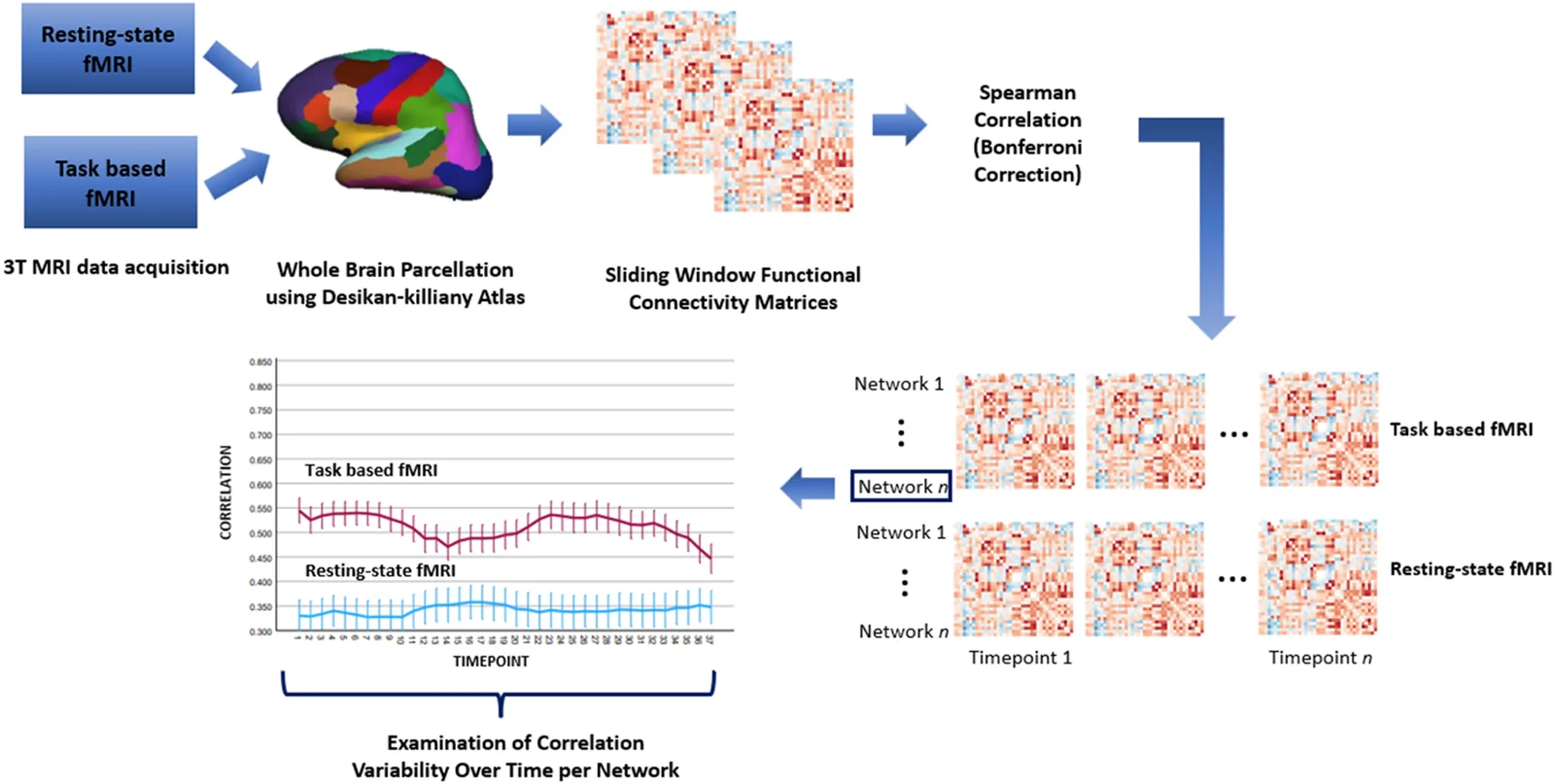
Schematic Overview of Data Processing. For data both from rs-fMRI and tb-fMRI, Desikan parcellation was overlaid on the pre-processed functional images to extract the corresponding time-series for each region. Sliding scale time series correlation was used to analyze dynamic FC time series data. Spearman correlation with Bonferroni correction was employed to identify network correlations over time. Linear mixed models with error and intercept random effects in the left and right hemispheric and inter-hemispheric networks

Language specificity is not defined a priori by the presence of rest–task differences. Instead, our analyses test whether state-dependent modulation of dynamic functional connectivity emerges as a distinguishing functional property across large-scale networks. Consequently, network classification is interpretive and post hoc, rather than defining a criterion used to select networks. Additionally, previous studies often use pre-determined parcellations to determine FC, and the dynamics of FC have been examined only within the pre-determined networks (Iraji et al. 2020). However, the language network is known to be interconnected to non-language networks such as regions responsible for cognition. Thus, in order to capture the complete FC during language processing, we used whole brain parcellations for the current study (Fedorenko et al. 2024b; Finn et al. 2014). Figure 1 shows the schematic overview of data processing.

MRI Preprocessing: Anatomical data were preprocessed to achieve accurate surface segmentation(RaviPrakash et al. 2021). More specifically, surface parcellation was performed using T1 weighted magnetization prepared rapid gradient echo Imaging (MPRAGE) and fluid-attenuated inversion recovery (FLAIR) images using FreeSurfer (Fischl 2012). The MPRAGE volume was registered with the MNI-305 atlas using AFNI registration for the cortical surface parcellation, followed by bias-field correction and skull stripping. Then, hemispheres were separated using a cutting-planes approach, and an initial white surface was generated for each hemisphere and then surface segmentations for each hemisphere were generated by following the intensity gradients between the white-matter and gray-matter. The pial surface using intensity gradients between the gray matter and cerebral spinal fluid was generated. The different contrast in the T2 weighted FLAIR images was used to refine the pial surface segmentation, after which surface labeling was conducted as described in Desikan et al. (2006) (Desikan et al. 2006).

fMRI Preprocessing: Structural and functional MRI data were preprocessed using Analysis of Functional NeuroImages toolbox (AFNI; version AFNI_21.2.06). High-resolution T1-weighted images were used for anatomical reference, tissue segmentation, functional–anatomical alignment, and mapping Desikan–Killiany atlas regions (34 cortical ROIs per hemisphere) onto functional data. Resting-state and task-based fMRI were processed using an identical pipeline across conditions. The first 7 volumes of each run were discarded, followed by despiking, rigid-body motion correction (6 parameters), and alignment of EPI data to individual anatomy using a local Pearson correlation cost function with visual quality checks. Functional data were nonlinearly normalized to MNI152 space via the anatomical image, and all analyses were conducted in standard space using identical transforms across conditions. No additional spatial smoothing was applied to preserve regional specificity for ROI-based connectivity analyses. Nuisance regression included motion parameters, white-matter principal components (8 ROIPC regressors), and low-frequency polynomial trends (polort = 3), with temporal band-pass filtering applied during regression; global signal regression was not performed. Motion was quantified using framewise displacement and Euclidean norm metrics, with a censoring threshold of 0.3 mm; no volumes exceeded this threshold in the final sample (0% censored), and mean motion was low (≈ 0.05 mm per TR). All runs underwent visual and quantitative quality control using AFNI’s @ss_review_basic outputs, and only participants with complete resting-state and task fMRI data and acceptable preprocessing and motion quality were included (Cox 1996).

Functional Connectivity Network Analysis: In order to generate the task network, first, the time-series of all task trials/blocks in a single participant were concatenated. Then, Pearson’s correlation was computed to generate the static FC. The associated *p*-values were corrected using Bonferroni correction. All significant common connections with *p*-values < 0.05 after correction were included in our analyses. We defined common connections as networks that were found in at least 85% of participants, in order to ensure that the networks included in our analyses were robust and reproducible. The participation threshold of 85% was determined empirically by first setting the threshold at 100% and systemically reducing the threshold by increments of 5%. A 100% threshold resulted in only a few connections. However, a threshold of 85% struck a balance between reproducibility and sensitivity, minimizing the risk of overlooking functionally meaningful connections that may vary slightly across individuals.

For dynamic functional connectivity (dFC) analyses, a sliding-window correlation approach was applied to ROI time series for both pea-state and task-based fMRI. Windows were defined as 42 consecutive TRs (corresponding to approximately 30 s, given TR = 0.72 s), with a step size of 3 TRs between successive windows, yielding 37 overlapping windows (timepoints) for task-based fMRI. This window length was selected to balance temporal resolution with stable estimation of correlation coefficients, consistent with prior dFC studies using task-based fMRI. Within each window, pairwise functional connectivity between ROIs was computed using Spearman correlation to reduce sensitivity to outliers and non-normality in short-window time series, followed by Bonferroni correction for multiple comparisons. Although the number of sliding windows derived from task-based fMRI is necessarily limited by task duration, statistical inference in the present study was performed at the group level using linear mixed-effects models. This approach leverages repeated measurements across participants rather than relying on the stability of individual-level trajectories. In addition, the use of a coarse-grained anatomical parcellation (Desikan–Killiany atlas) reduces estimation variance within each window, further supporting the robustness of the dynamic connectivity estimates.

### Statistical analyses

Linear mixed-effect models with random intercept and residual error were used to test linear trend over time in the common network connections in intra-hemispheric (left and right hemisphere) and inter-hemispheric networks, with time (i.e. 37 timepoints both for rs-fMRI and tb-fMRI) as the main effect. Given that the rs-fMRI acquisition time was longer than that for tb-fMRI, we conducted a sensitivity analysis to ensure the consistency of BOLD signal patterns acquired at later times during the rs-fMRI scan. Models were adjusted for age and sex effects. Linear mixed-effect models with random intercept and residual error were also used to directly compare linear trend over time between rs-fMRI and tb-fMRI in the same network connections, with time (i.e., 37 timepoints both for rs-fMRI and tb-fMRI) as fixed effect. Models were adjusted for repeated measures, within-subject correlations, and the subject characteristics of age and sex.

Using the formula; ${\mathrm{y}}\, = \,{\mathrm{X}}\beta \, + \,{\mathrm{Zb}}\, + \,{\mathrm{e}}$

Where y is the response variable representing the correlation values in all 37 timepoints, Xβ represents the fixed effects (sex, Age, and confounding factors), Zb represents random effects for the subjects, and e denotes residual errors.

A *p*-value of **<** 0.01 was considered statistically significant for both models to account for the multiple comparisons performed in the analyses Additionally, descriptive statistics, Fisher’s exact test, chi-square tests, and the Wilcoxon rank sum test were used to describe participants’ demographics as well as the patterns of correlation strength (mean Fischer z-transformed functional connectivity values across sliding windows). As mentioned above, to ensure network robustness and reproducibility, we included only common networks that were activated in 85% of all participants in our analyses. This was determined empirically. No correlation strength threshold was applied.

To ensure comparability across conditions, only the first 37 sliding windows of the rs-fMRI data were analyzed, matching the number of windows obtained from tb-fMRI. A sensitivity analysis comparing early versus late resting-state windows confirmed no systematic differences in network dynamics. To assess the robustness of dynamic functional connectivity estimates to windowing parameters, sensitivity analyses were performed using multiple sliding-window configurations, including window lengths of 42 TRs with step sizes of 3 and 6 TRs (Win42_Stride_3; Win42_Stride_6). For each configuration, subject-level windowed functional connectivity matrices were computed independently, and all subsequent group-level analyses were repeated using identical statistical models. This approach allowed evaluation of whether observed temporal trends and state-dependent effects were consistent across reasonable choices of window overlap and temporal sampling.

Additionally, a Friedman test was used to assess whether correlations tightened over time, as indicated by reductions in standard deviation, while a Wilcoxon signed-rank test was used to evaluate increases in overall correlation strength, measured by changes in mean correlation, in left, right, and interhemispheric networks. The high value is an indication of a significant increase in the mean correlation values.

A power analysis determined that our study has a high statistical power with a 95% confidence interval of 96–100.

## Results

### Behavioral result

The accuracy of the story task was 95.57 ± 7.57, indicating that the majority of the participants were highly engaged and performed well on the task.

### Temporal linear trends in dynamic functional connectivity

**Fig. 2:**
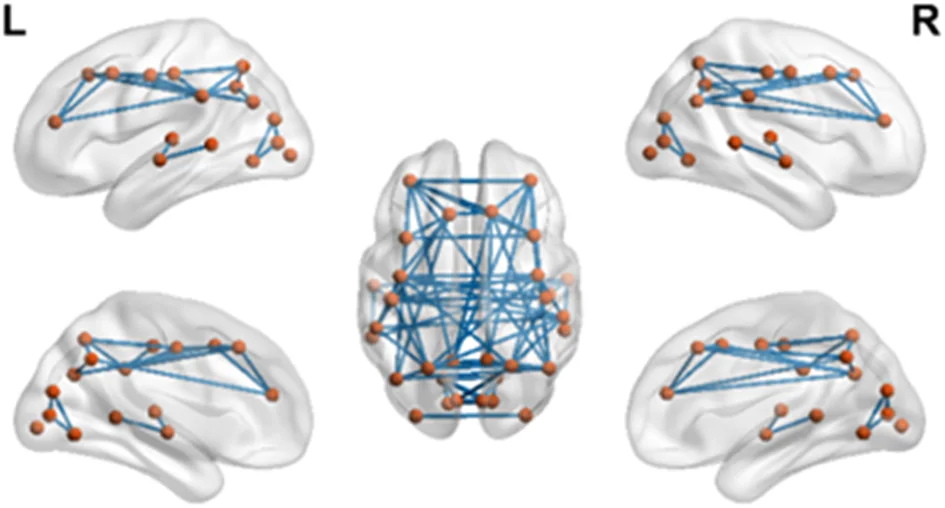
Common Network Connections during Language Processing. 40 intra-hemispheric network connections (20 common left hemispheric network connections + 20 common right hemispheric networks), and 35 inter-hemispheric network connections were identified in dynamic rs-fMRI

There were 40 intra-hemispheric networks (i.e., 20 common left hemispheric network connections, 20 common right hemispheric networks) and 35 inter-hemispheric networks identified in dynamic rs-fMRI, respectively (Fig. 2). Additionally, 77 common networks were identified during tb-fMRI. There were 42 intra-hemispheric network connections (i.e., 21 common left hemispheric networks and 21 common right hemispheric networks) and 35 inter-hemispheric networks identified, respectively. In order to evaluate the differences in dynamic FC between rs-fMRI and tb-fMRI, we analyzed the dynamic FC characteristics of the same networks found in both rs-fMRI and tb-fMRI. Therefore, the two connections/edges that were only identified in tb-fMRI (i.e., left superior frontal ‒ left supramarginal connection and right postcentral ‒ right supramarginal connection) were not included in the analyses.

**Fig. 3:**
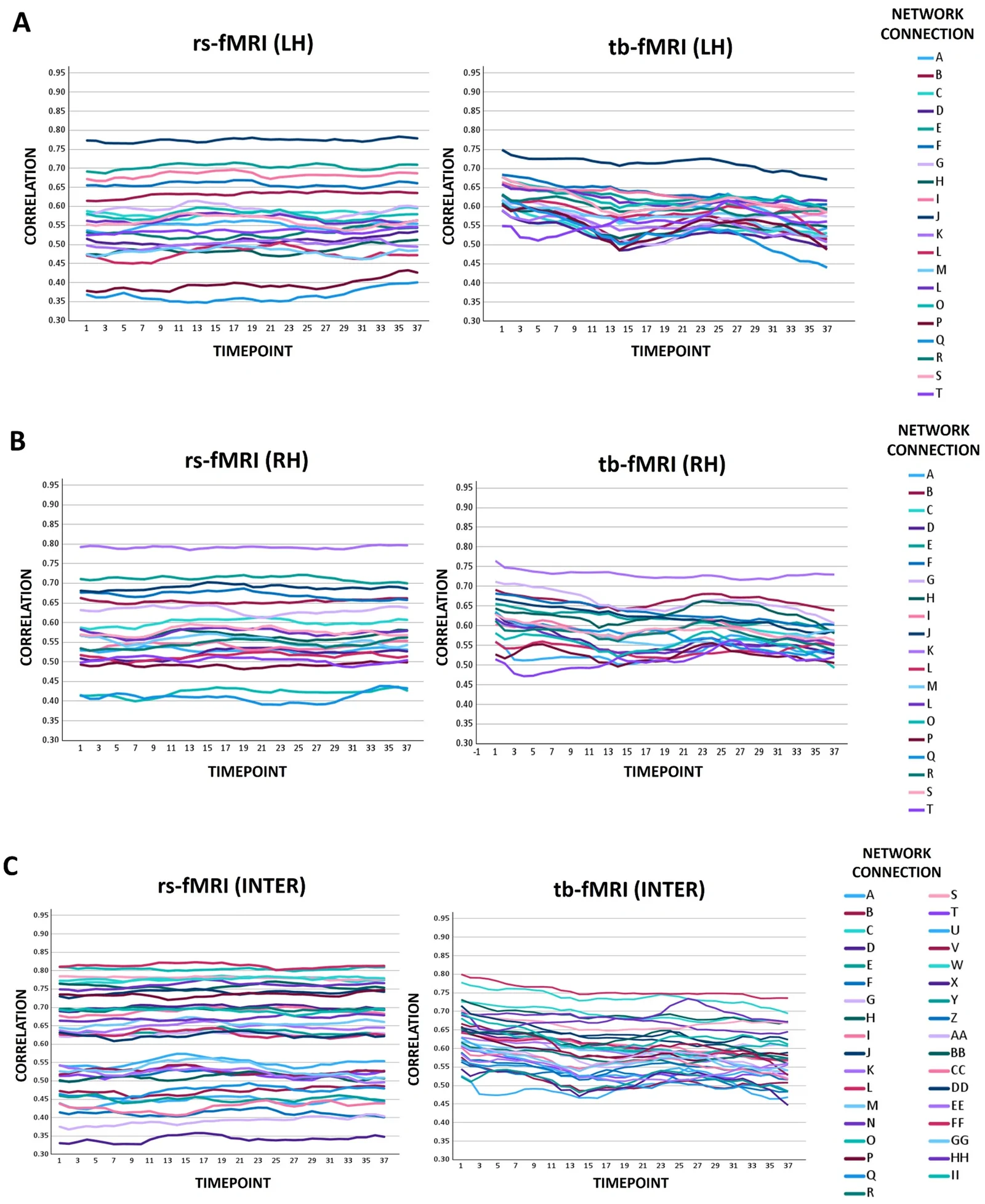
Mean Linear trend over time averaged across network connections: **A** Left hemispheric connections in rs-fMRI vs. tb-fMRI; **B** Right hemispheric connections in rs-fMRI vs. tb-fMRI, and **C** Inter-hemispheric connections in rs-fMRI vs. tb-fMRI. Correlation strengths were spread across a wide variability range in all connections in rs-fMRI. On the contrary, there was increased correlation strength in the left and right hemispheric connections, and a tightening of correlations over time in all connections in tb-fMRI. *Left and Right Hemispheric connections*: A: Banks of superior temporal sulcus—Superior temporal; B:Caudal middle frontal—Inferior parietal; C:Caudal middle frontal—Rostral middle frontal; D: Caudal middle frontal—Superior frontal; E: Cuneus—Lingual; F: Cuneus—Pericalcarine; G: Inferior parietal—Precuneus; H: Inferior parietal—Superior frontal; I: Lingual—Pericalcarine; J: Postcentral—Precentral; K: Postcentral—Superior parietal; L: Postcentral—Supramarginal; M: Precentral—Superior frontal; N: Precentral—Superior parietal; O: Precentral—Supramarginal; P: Precuneus—Rostral middle frontal; Q: Precuneus—Superior parietal; R: Rostral middle frontal—Superior frontal; S: Superior parietal—Supramarginal; T: Superior temporal—Transverse temporal. *Inter-hemispheric connections*: A: Banks of Superior Temporal—Sulcus Superior Temporal; B: Caudal Middle Frontal—Caudal Middle Frontal; C:Cuneus—Cuneus; D: Cuneus—Lingual; E: Cuneus—Pericalcarine; F: Cuneus—Superior Parietal; G: Inferior Parietal—Inferior Parietal; H: Lateral Occipital—Lateral Occipital; I: Lingual—Cuneus; J: Lingual—Lingual; K: Lingual—Pericalcarine; L: Pericalcarine—Cuneus; M: Pericalcarine—Lingual; N: Pericalcarine—Pericalcarine; O: Postcentral—Postcentral; P: Postcentral—Precentral; Q: Postcentral—Supramarginal; R: Precentral—Postcentral; S: Precentral—Precentral; T: Precentral Superior Parietal; U: Precentral—Supramarginal; V: Precuneus—Inferior Parietal; W: Precuneus—Precuneus; X: Precuneus—Rostral Middle Frontal; Y: Precuneus—Superior Frontal; Z: Rostral Middle—Frontal Caudal Middle Frontal; AA: Rostral Middle Frontal—Inferior Parietal; BB: Rostral Middle—Frontal Rostral Middle Frontal; CC: Rostral Middle Frontal—Superior Frontal; DD: Superior Frontal—Superior Frontal; EE: Superior Parietal—Precentral; FF: Superior Parietal—Superior Parietal; GG: Superior Parietal—Supramarginal; HH: Superior Temporal—Superior Temporal; II Supramarginal—Supramarginal

**Fig. 4:**
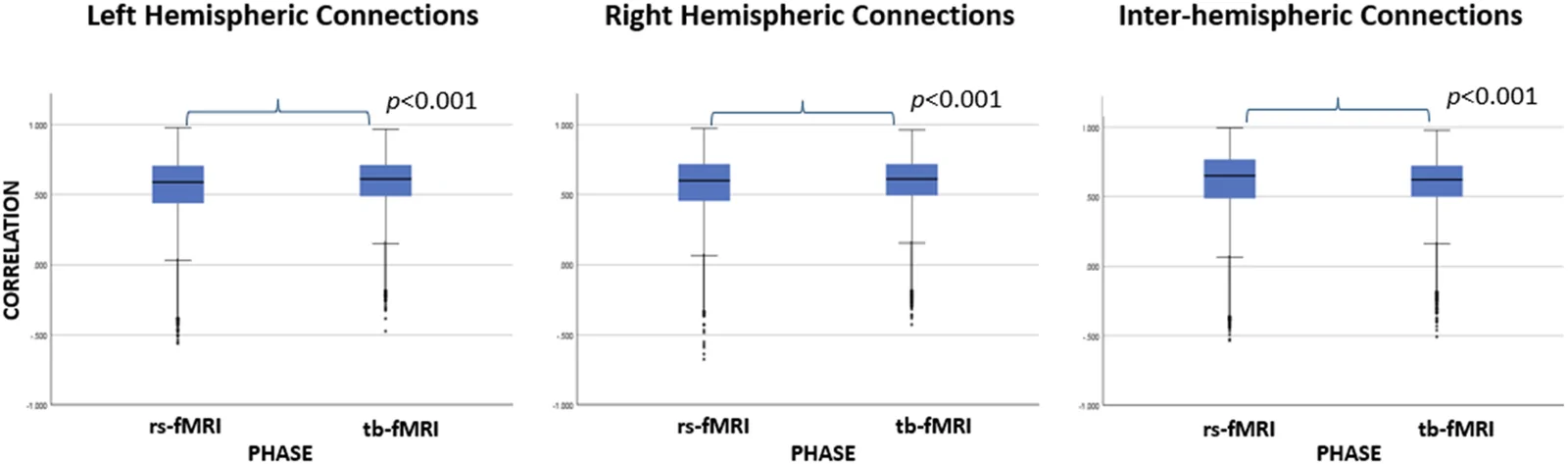
Boxplots of mean Linear trend over time in rs-fMRI and tb-fMRI. There was an increase in overall correlation strength (as measured by mean correlation difference) in the left and right hemispheric connections, and decreased strength in inter-hemispheric connections. There was a tightening of correlations over time (as measured by SD reduction) in all network connections

Linear mixed models directly compared linear trend differences between dynamic rs-fMRI and tb-fMRI. Variability of the correlations (variance or standard deviation) of Fisher z-transformed dynamic FC values across windows within a condition) over time for all networks (i.e., left and right hemispheric connections and inter hemispheric connections) except for two connections/edges (i.e., right bank of superior temporal sulcus – right superior temporal connection and right superior temporal – right transverse temporal connection) was substantially different between rs-fMRI and tb-fMRI, accounting for repeated measures, within subject correlations, and age and sex (all *p* < 0.001; Table 2). More specifically, correlation strengths during the resting-state were spread across a wide range in left and right hemispheric connections, as well as in inter-hemispheric connections whereas tb-FC correlation strengths centered around a condensed range (left hemispheric connections: median = 0.61 [IQR 0.486–0.710]; right hemispheric connections: median = 0.611 [IQR = 0.491–0.715]; inter-hemispheric connections: median = 0.622 [IQR = 0.498–0.726]). There was a tightening of correlations over time (as measured by SD reduction) in left hemispheric network connections (SD = 0.168; 17.81% SD decrease; *χ*^*2*^(1) = 816.157; *p* < 0.001), right hemispheric connections (SD = 0.167; 14.08% SD decrease; *χ*^*2*^(1) = 145.716; *p* < 0.001) and inter-hemispheric connections (SD = 0.170; 18.93% SD decrease; *χ*^*2*^(1) = 1169.65; *p* < 0.001) (Figs. 3A–C and 4). In addition, the linear trend during task-based condition demonstrated an increase in overall correlation strength in the left hemispheric connections (*mean* = 0.586; 5.5% increase as measured by mean correlation difference; z = − 39.44; *p* < 0.001) and right hemispheric connections (*mean* = 0.592; 2.8% increase; z = − 21.161; *p* < 0.001). There was decreased strength in inter-hemispheric connections (*mean* = 0.599; 0.9% decrease; z = − 27.441; *p* < 0.001).

**Table 2:** Direct comparison of the linear trend over time between dynamic resting-state functional connectivity networks and dynamic task-based functional connectivity networks

Hemisphere	Connections		rs-fMRI vs. tb-fMRI	
Left	ROI1	ROI2	*F*-value	*p*-value
1	Banks of superior temporal sulcus	Superior temporal	173.59	**< 0.001**
2	Caudal middle frontal	Inferior parietal	1025.87	**< 0.001**
3	Caudal middle frontal	Rostral middle frontal	292.38	**< 0.001**
4	Caudal middle frontal	Superior frontal	48.92	**< 0.001**
5	Cuneus	Lingual	1911.78	**< 0.001**
6	Cuneus	Pericalcarine	64.35	**< 0.001**
7	Inferior parietal	Precuneus	131.79	**< 0.001**
8	Inferior parietal	Superior frontal	687.2	**< 0.001**
9	Lingual	Pericalcarine	796.07	**< 0.001**
10	Postcentral	Precentral	1231.75	**< 0.001**
11	Postcentral	Superior parietal	309.03	**< 0.001**
12	Postcentral	Supramarginal	1886.67	**< 0.001**
13	Precentral	Superior frontal	1255.51	**< 0.001**
14	Precentral	Superior parietal	514.35	**< 0.001**
15	Precentral	Supramarginal	408.75	**< 0.001**
16	Precuneus	Rostral middle frontal	3019.86	**< 0.001**
17	Precuneus	Superior parietal	3170.86	**< 0.001**
18	Rostral middle frontal	Superior frontal	851.19	**< 0.001**
19	Superior parietal	Supramarginal	450.7	**< 0.001**
20	Superior temporal	Transverse temporal	117.74	**< 0.001**

Right	ROI1	ROI2		
1	Banks of Superior Temporal Sulcus	Superior Temporal	0.07	0.798
2	Caudal Middle Frontal	Inferior Parietal	15.22	**< 0.001**
3	Caudal Middle Frontal	Rostral Middle Frontal	14.39	**< 0.001**
4	Caudal Middle Frontal	Superior Frontal	290.03	**< 0.001**
5	Cuneus	Lingual	2531.9	**< 0.001**
6	Cuneus	Pericalcarine	240.96	**< 0.001**
7	Inferior Parietal	Precuneus	171.9	**< 0.001**
8	Inferior Parietal	Rostral Middle Frontal	680.72	**< 0.001**
9	Inferior Parietal	Superior Frontal	468	**< 0.001**
10	Lingual	Pericalcarine	1088.43	**< 0.001**
11	Postcentral	Precentral	1423.23	**< 0.001**
12	Postcentral	Superior Parietal	81.3	**< 0.001**
13	Precentral	Superior Parietal	54	**< 0.001**
14	Precentral	Supramarginal	45.54	**< 0.001**
15	Precuneus	Rostral Middle Frontal	2514.64	**< 0.001**
16	Precuneus	Superior Frontal	224.92	**< 0.001**
17	Precuneus	Superior Parietal	3608.96	**< 0.001**
18	Rostral Middle Frontal	Superior Frontal	309.5	**< 0.001**
19	Superior Parietal	Supramarginal	13.04	**< 0.001**
20	Superior Temporal	Transverse Temporal	5.99	0.014

Inter-Hemisphere	ROI1 (Left)	ROI2 (Right)		
1	Banks of Superior Temporal Sulcus	Superior Temporal	359.37	**< 0.001**
2	Caudal Middle Frontal	Caudal Middle Frontal	218.77	**< 0.001**
3	Cuneus	Cuneus	2826.06	**< 0.001**
4	Cuneus	Lingual	2444.54	**< 0.001**
5	Cuneus	Pericalcarine	110.03	**< 0.001**
6	Cuneus	Superior Parietal	10.49	**< 0.001**
7	Inferior Parietal	Inferior Parietal	94.54	**< 0.001**
8	Lateral Occipital	Lateral Occipital	1178.17	**< 0.001**
9	Lingual	Cuneus	1808.6	**< 0.001**
10	Lingual	Lingual	4590.23	**< 0.001**
11	Lingual	Pericalcarine	723.6	**< 0.001**
12	Pericalcarine	Cuneus	86.62	**< 0.001**
13	Pericalcarine	Lingual	1175.14	**< 0.001**
14	Pericalcarine	Pericalcarine	2329.75	**< 0.001**
15	Postcentral	Postcentral	9259.6	**< 0.001**
16	Postcentral	Precentral	4165.9	**< 0.001**
17	Postcentral	Supramarginal	337.14	**< 0.001**
18	Precentral	Postcentral	2873.98	**< 0.001**
19	Precentral	Precentral	4479.5	**< 0.001**
20	Precentral	Superior Parietal	75.03	**< 0.001**
21	Precentral	Supramarginal	0.11	**< 0.001**
22	Precuneus	Inferior Parietal	930.65	**< 0.001**
23	Precuneus	Precuneus	616.15	**< 0.001**
24	Precuneus	Rostral Middle Frontal	3475.61	**< 0.001**
25	Precuneus	Superior Frontal	711.56	**< 0.001**
26	Rostral Middle Frontal	Caudal Middle Frontal	2428.23	**< 0.001**
27	Rostral Middle Frontal	Inferior Parietal	3971.4	**< 0.001**
28	Rostral Middle Frontal	Rostral Middle Frontal	2513.39	**< 0.001**
29	Rostral Middle Frontal	Superior Frontal	2742.92	**< 0.001**
30	Superior Frontal	Superior Frontal	135.34	**< 0.001**
31	Superior Parietal	Precentral	41.87	**< 0.001**
32	Superior Parietal	Superior Parietal	1513.96	**< 0.001**
33	Superior Parietal	Supramarginal	152.41	**< 0.001**
34	Superior Temporal	Superior Temporal	37.41	**< 0.001**
35	Supramarginal	Supramarginal	913.04	**< 0.001**

Results are from repeated measures models that tested for time effect and adjusted for age, sex, where applicable

### Patterns of dynamic functional connectivity across states

Among these 75 common networks identified during rs-fMRI, the dynamic FC showed no significant linear trend over time on any of the left-hemispheric networks (*p* > 0.12) nor right hemisphere networks (*p* > 0.11), accounting for age and sex. Similarly, there was no linear trend over time on any of the inter-hemispheric networks (*p* > 0.14), accounting for age and sex during rs-fMRI (Table 3).

**Table 3:** Linear trend over time in dynamic resting-state functional connectivity networks and dynamic task-based functional connectivity networks

Hemisphere	Connections			rs-fMRI		tb-fMRI	
Left	ROI1		ROI2	*F*-value	*p*-value	*F*-value	*p*-value
1	Banks of superior temporal sulcus		Superior temporal	0.47	0.49	0.74	0.39
2	Caudal middle frontal		Inferior parietal	1.62	0.2	10.62	**0.001**
3	Caudal middle frontal		Rostral middle frontal	0.87	0.35	5.05	0.026
4	Caudal middle frontal		Superior frontal	2.05	0.15	8.5	**0.004**
5	Cuneus		Lingual	0.31	0.58	10.37	**0.002**
6	Cuneus		Pericalcarine	0.02	0.9	21.52	**< 0.001**
7	Inferior parietal		Precuneus	0.42	0.52	5.9	0.016
8	Inferior parietal		Superior frontal	1.93	0.17	6.29	0.013
9	Lingual		Pericalcarine	0.34	0.56	12.98	**< 0.001**
10	Postcentral		Precentral	0.67	0.41	7.53	**0.007**
11	Postcentral		Superior parietal	0.53	0.47	7.52	**0.007**
12	Postcentral		Supramarginal	0.37	0.54	7.41	**0.007**
13	Precentral		Superior frontal	0.13	0.72	7.72	**0.006**
14	Precentral		Superior parietal	0.05	0.82	6.75	0.01
15	Precentral		Supramarginal	0.06	0.81	9.96	**0.002**
16	Precuneus		Rostral middle frontal	2.41	0.12	7.06	**0.009**
17	Precuneus		Superior parietal	2.01	0.16	44.8	**< 0.001**
18	Rostral middle frontal		Superior frontal	1.9	0.17	2.95	0.088
19	Superior parietal		Supramarginal	0.8	0.37	13.22	**< 0.001**
20	Superior temporal		Transverse temporal	0.38	0.54	8.11	**0.005**

Right	ROI1		ROI2				
1	Banks of Superior Temporal Sulcus		Superior Temporal	0.02	0.9	0.26	0.61
2	Caudal Middle Frontal		Inferior Parietal	0.2	0.65	0.2	0.65
3	Caudal Middle Frontal		Rostral Middle Frontal	0.53	0.47	0.79	0.37
4	Caudal Middle Frontal		Superior Frontal	2.63	0.11	11.37	**0.001**
5	Cuneus		Lingual	0.46	0.5	6.7	0.01
6	Cuneus		Pericalcarine	1.91	0.17	24.12	**< 0.001**
7	Inferior Parietal		Precuneus	0.14	0.71	20.4	**< 0.001**
8	Inferior Parietal		Rostral Middle Frontal	0.01	0.94	0.57	0.45
9	Inferior Parietal		Superior Frontal	0.03	0.87	1.27	0.26
10	Lingual		Pericalcarine	0.2	0.65	16.31	**< 0.001**
11	Postcentral		Precentral	0.07	0.79	0.26	0.61
12	Postcentral		Superior Parietal	0.42	0.52	0.08	0.78
13	Precentral		Superior Parietal	0.96	0.33	1.87	0.17
14	Precentral		Supramarginal	0.11	0.74	4.83	0.029
15	Precuneus		Rostral Middle Frontal	0.78	0.38	1.52	0.22
16	Precuneus		Superior Frontal	0.09	0.77	0.22	0.64
17	Precuneus		Superior Parietal	0.12	0.73	13.21	**< 0.001**
18	Rostral Middle Frontal		Superior Frontal	1.23	0.27	0.5	0.48
19	Superior Parietal		Supramarginal	0.01	0.92	5.02	0.026
20	Superior Temporal		Transverse Temporal	0.87	0.35	10.19	**0.002**

Inter-hemisphere	ROI1 (LEFT)		ROI2 (RIGHT)				
1	Banks of superior temporal sulcus		Superior Temporal	0.68	0.41	0.05	0.82
2	Caudal Middle Frontal		Caudal Middle Frontal	1.4	0.24	4.18	0.042
3	Cuneus		Cuneus	1.17	0.28	21.44	**< 0.001**
4	Cuneus		Lingual	0.07	0.79	14.79	**< 0.001**
5	Cuneus		Pericalcarine	0.00	0.99	24.83	**< 0.001**
6	Cuneus		Superior Parietal	0.65	0.42	4.11	0.044
7	Inferior Parietal		Inferior Parietal	0.00	0.96	4.22	0.041
8	Lateral Occipital		Lateral Occipital	0.01	0.94	2.58	0.11
9	Lingual		Cuneus	1.04	0.31	9.4	**0.003**
10	Lingual		Lingual	0.75	0.39	11.45	**0.001**
11	Lingual		Pericalcarine	0.19	0.66	18.48	**< 0.001**
12	Pericalcarine		Cuneus	0.39	0.53	12.08	**0.001**
13	Pericalcarine		Lingual	0.35	0.56	9.82	**0.002**
14	Pericalcarine		Pericalcarine	1.63	0.2	9.25	**0.003**
15	Postcentral		Postcentral	0.00	0.96	8.98	**0.003**
16	Postcentral		Precentral	0.43	0.51	6.38	0.012
17	Postcentral		Supramarginal	1.43	0.23	9.48	**0.002**
18	Precentral		Postcentral	0.01	0.92	1.9	0.17
19	Precentral		Precentral	0.62	0.43	2.93	0.089
20	Precentral		Superior Parietal	0.03	0.86	12.04	**0.001**
21	Precentral		Supramarginal	0.02	0.88	13.71	**< 0.001**
22	Precuneus		Inferior Parietal	0.24	0.62	23.84	**< 0.001**
23	Precuneus		Precuneus	0.7	0.41	17.01	**< 0.001**
24	Precuneus		Rostral Middle Frontal	0.26	0.61	2.32	0.13
25	Precuneus		Superior Frontal	0.00	0.94	0.38	0.54
26	Rostral Middle Frontal		Caudal Middle Frontal	0.01	0.92	0.35	0.56
27	Rostral Middle Frontal		Inferior Parietal	1.72	0.19	1.97	0.16
28	Rostral Middle Frontal		Rostral Middle Frontal	0.05	0.83	5.09	0.025
29	Rostral Middle Frontal		Superior Frontal	1.33	0.25	4.54	0.034
30	Superior Frontal		Superior Frontal	0.03	0.87	15.51	**< 0.001**
31	Superior Parietal		Precentral	2.21	0.14	2.77	0.1
32	Superior Parietal		Superior Parietal	0.4	0.53	18.3	**< 0.001**
33	Superior Parietal		Supramarginal	1.4	0.24	5.25	0.023
34	Superior Temporal		Superior Temporal	1.71	0.19	4.76	0.03
35	Supramarginal		Supramarginal	0.34	0.56	9.65	**0.002**

Results are from repeated measures models that tested for time effect and adjusted for age, sex, where applicable

**Fig. 5:**
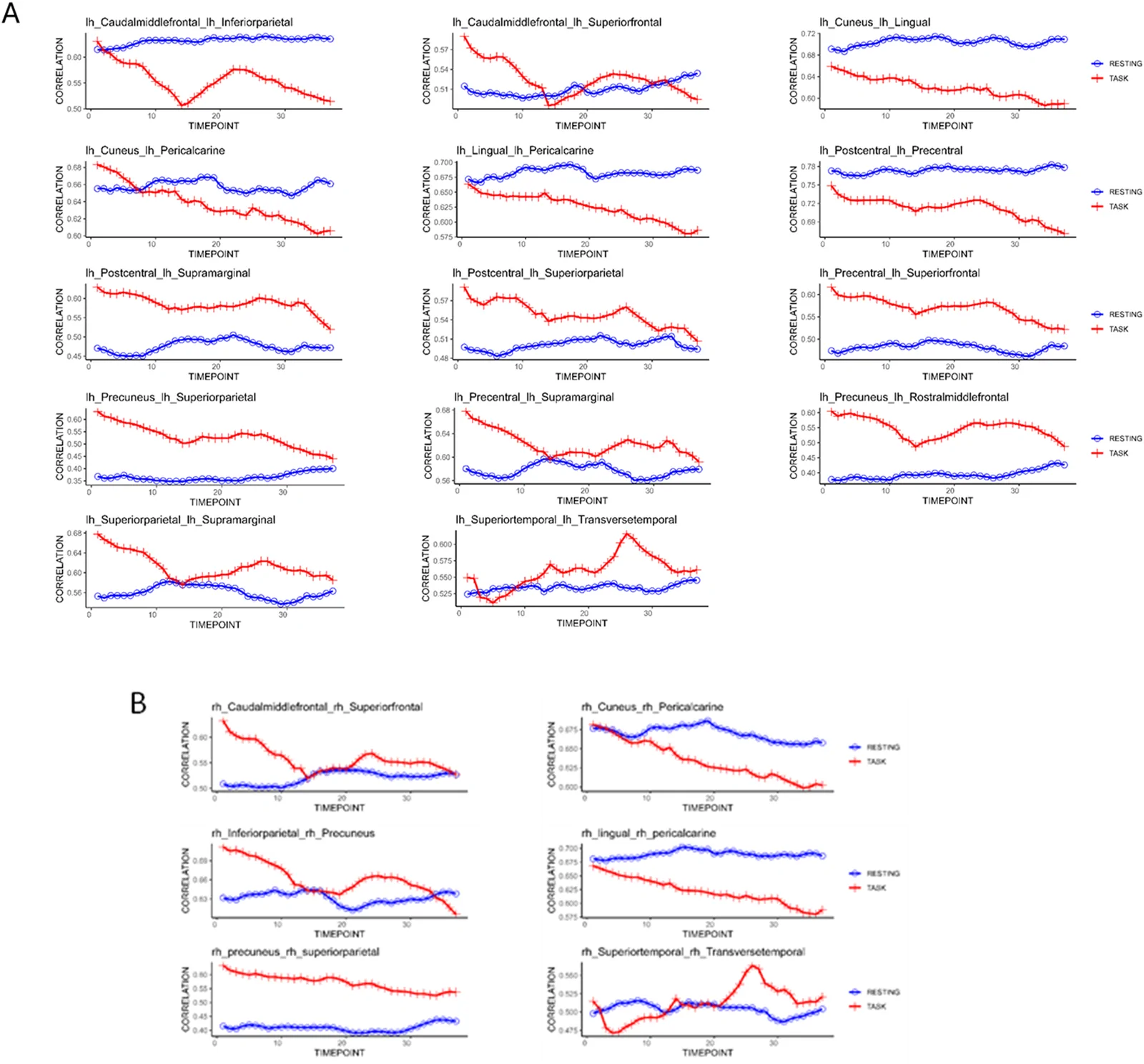

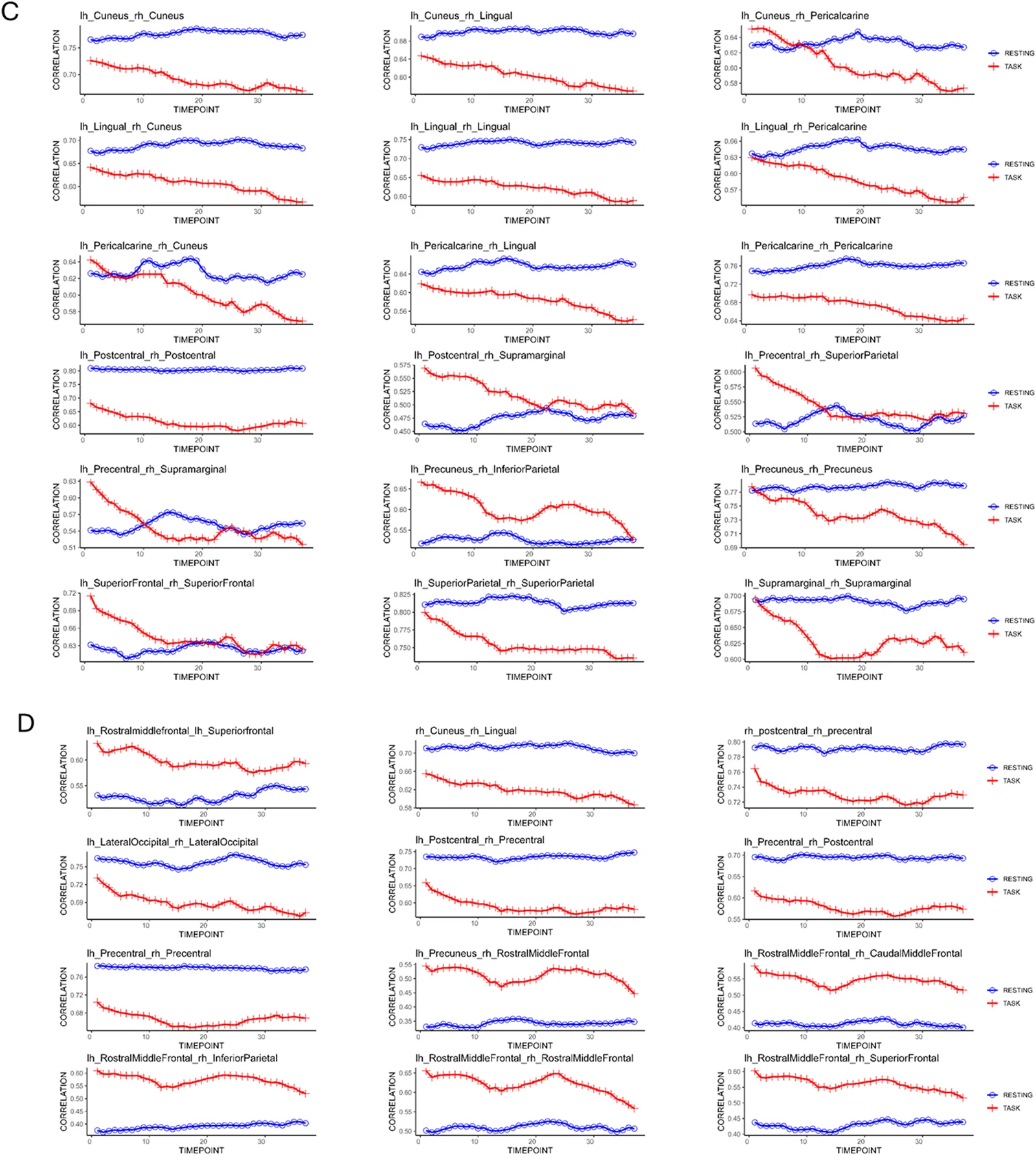
Dynamic task-based network connections averaged across subjects that showed linear trend over time distinctly different from dynamic resting-state network pattern. **A** Left hemispheric network connections; Right hemispheric network connections; **C** Inter-hemispheric network connections; and **D** Network connections that demonstrate patterns that parallel resting-state networks. There were dynamic task-based networks that showed linear trend over time distinctly different from dynamic rs-fMRI networks. These networks were likely language specific. Other networks demonstrated patterns that parallel resting-state networks with similar dynamic correlation variability patterns observed during rs-fMRI. These networks likely represent language-related cognitive processing

In contrast, dynamic FC during tb-fMRI showed marked linear trend over time. Among the 75 common networks in dynamic tb-fMRI, linear trend over time was observed in 70% of the common networks in the left hemisphere, accounting for age and sex effects (*p* < 0.001; Table 3). Among the right hemispheric networks in tb-fMRI, the time effect was observed on 30% of the common networks, accounting for age and sex effects (*p* < 0.001). Although the same networks were analyzed in both rs- and tb-fMRI, more networks showed different temporal linear trend over time patterns in the left hemisphere (70%) than in the right hemisphere (30%) for dynamic rs-FC vs. dynamic tb-FC. A similar pattern was found in inter-hemispheric common networks in tb-fMRI; 37% of the common networks showed linear trend over time (*p* < 0.001; Fig. 5), where only 0% of those same networks showed such a pattern during dynamic rs-FC.

On the contrary, there were dynamic task-based networks (6 left hemispheric networks, 14 right hemispheric networks, and 16 inter-hemispheric networks, respectively) that demonstrated no statistically significant linear trend patterns over time (*p* > 0.02; Table 3). Notably, several dynamic task-based networks demonstrated both increasing and decreasing patterns that counterbalanced each other, resulting in no statistically significant linear trend over time. After excluding those networks, the remaining dynamic task-based networks showed linear trend patterns over time that aligned with those observed in resting-state networks (Fig. 5). These networks likely represent language-related cognitive processing, such as generalized attentional, saliency, and default mode information processing that are subserving language processing.

## Discussion

### Temporal linear trends in dynamic functional connectivity

We tested whether the patterns of FC network dynamics differentiate the functional specialization of each network during language processing. To this end, we first tested whether the dynamic functional connectivity network linear trend over time within the same networks differed between rs-fMRI and tb-fMRI during language processing in a large cohort of healthy participants. Our analyses confirmed differences in the temporal dynamics of neural networks during auditory language comprehension between dynamic rs-fMRI and dynamic tb-fMRI. Direct comparison of the linear trend over time between dynamic rs-fMRI and tb-fMRI indicated that there were substantial differences in temporal behavior within the same networks. All left hemispheric network connections, all right hemispheric network connections except for two connections, and inter-hemispheric network connections showed differences in FC linear trend over time between rs-fMRI and tb-fMRI. More specifically, there was no variability identified in any of the intra- and inter-hemispheric network connections in dynamic rs-fMRI. However, in dynamic tb-fMRI, specific networks demonstrated increased FC variability over time These results confirmed that there is task-related temporal FC linear trend over time during language processing.

The results of the temporal FC variability in tb-fMRI agree with the current neural models of speech and language processing, which embody both spatial and temporal dynamics of cortical neuronal activity across large scale neural network across the whole brain (Canolty et al. 2007; Hickok and Poeppel 2007; Jarret et al. 2022; Poeppel and Assaneo 2020). Multi-scale processing model and asymmetric sampling in time theory suggest that acoustic signals contain critical temporal information, such as segmental and syllabic information, and language processing relies on this information. Depending on the temporal information that acoustic signals convey, acoustic signals (both speech and non-speech) are simultaneously processed at multiple different timescales asymmetrically in both left and right hemispheres (Poeppel 2003; Poeppel and Assaneo 2020). The temporal components are inherent to language processing, especially in the processing of rapidly changing auditory signals during language comprehension. For this reason, the dynamics of FC are essential elements for understanding language processing and, more specifically for differentiating linguistic information processing from attentional and other general cognitive information processing.

Both animal and human research suggest that temporal dynamics of FC originate from neuronal activations, instead of random fluctuations caused by physiological noise (Matsui et al. 2019; Tagliazucchi et al. 2012). Temporal FC variability reflects cognitive processes (Betzel et al. 2020; Hutchison et al. 2013; Kucyi and Davis 2014; Kurtin et al. 2023; Song and Lee 2023). In particular, FC in tb-fMRI reflects task-related neural networks and better captures individual differences during tb-fMRI. Given this fact, we think that tb-fMRI may demonstrate greater test-retest reliability and prediction accuracy (Greene et al. 2018; Jiang et al. 2020; Kristo et al. 2014; Rosazza et al. 2014; Tuominen et al. 2023). This may explain why the FC network linear trend over time was observed only during tb-fMRI.

Assessing linear trend strengths over time demonstrated distinct pattern differences in the left and right hemispheric connections (namely that overall correlation strength increased, whereas it decreased in strength in inter-hemispheric connections during tb-fMRI). This variability was not seen in any of the networks in either hemisphere during rs-fMRI. There also was a tightening of correlations over time, as measured by standard deviation reduction, around the new mean in all network connections observed only in tb-fMRI. Di and colleagues (2013) suggested that, with increased efficiency, the whole brain shows less segregation as independent modules during cognitive tasks compared to rs-fMRI (Di et al. 2013). The observed increased correlation strength and tightening of correlations over time during language processing in tb-fMRI in the current study also may be a reflection of increased efficiency during the engagement of language tasks (Gonzalez-Castillo and Bandettini 2018).

Previous studies that tested dynamic FC during cognitive tasks reported task-induced FC correlation variability reduction during cognitive tasks, such as a visual classification task and a two-back working memory task in some of the networks in healthy adults (Chen et al. 2015; Elton and Gao 2015). For instance, there was a task-induced reduction in FC variability during a visual classification task within and between the dorsal attention network, the default-mode network, the salience network, and the executive control network in healthy participants. In the same study, there was task-induced FC variability increase within visual and somatosensory networks (Elton and Gao 2015). Another study with a two-back working memory task in healthy participants also reported reduced FC variability during the task over the 1-min sliding window correlations between the default network and executive-control network and each brain voxel (Chen et al. 2015). However, in the current study, an increased linear trend over time was observed in tb-fMRI. Although there are some methodological differences between these studies and the current study, the differences in dynamic correlation patterns may be an indication that brain integration may be task dependent(Shine et al. 2016) and that dynamic integration patterns during language processing are language specific and are uniquely different from those associated with general cognitive encoding processing.

### Patterns of dynamic functional connectivity across states

We then tested whether the patterns of network dynamics differentiate the functional specialization of each network during language processing. We hypothesized that language-specific networks showed task-related temporal FC linear trend over time, whereas networks processing general cognitive information may follow rs-fMRI dynamics. The results of the current study show that, during language processing, some networks showed FC linear trend over time that markedly differs from the temporal linear trend patterns observed during the resting-state. Indeed, 70% of the left-hemispheric network connections, 30% of the right-hemispheric network connections, and 37% of the inter-hemispheric network connections showed linear trend over time distinctly different from rs-fMRI dynamics. These dynamic task-based networks that showed distinct linear trend pattern differences from those of dynamic rs-FC may be thought of as being language-specific.

On the contrary, the other dynamic task-based networks, mostly in the right hemisphere and inter-hemispheric networks, showed no significant linear trend patterns differences from those during the resting-state. Among them, several dynamic task-based networks demonstrated linear trend patterns over time that parallel resting-state networks. Previous studies have reported that resting-state networks are likely processing generalized attentional, saliency, and default mode information processing that are subserving language processing, i.e., language-related cognitive processing (Fedorenko et al. 2011, 2024a; Geranmayeh et al. 2014; Mineroff et al. 2018; Wylie and Regner 2014). Hence, we think that networks that parallel resting-state networks are likely to process similar types of information related to cognitive processing. The networks that demonstrated differing dynamics from the resting-state pattern may be processing more language-specific information, although we are limited in providing the precise linguistic element that is being processed. These results suggest that dynamic FC analyses of task-based fMRI may shed light, in real-time, on the interplay between language-specific networks and language-related networks and may enable the identification of the type of information processing across large-scale neural networks without cognitive localizer tasks in the human brain.

Previous functional region of interest (ROI) studies typically employed language localizer tasks along with cognitive localizer tasks (e.g., a working memory task) to identify functional dissociation between language-specific regions and language-related regions. (Blank et al. 2014; I. A. Blank and Fedorenko 2017; Fedorenko, 2014). For instance, Blank and Fedorenko (2017) employed a story reading task and a spatial working memory task to localize both language-specific regions and language-related cognitive regions (i.e., multiple-demand network) and then compared BOLD signal time courses (I. A. Blank and Fedorenko 2017). By employing a story reading task and a spatial working memory task, Mineroff, Blank, & Fedorenko (2018) further identified dissociation between the language-specific networks and the multiple-demand network as well as the DMN (Mineroff, Blank, & Fedorenko, 2018). Another functional ROI study measured pairwise correlation between time courses of regions during the brain’s resting-state and regions activated during a spatial working memory to identify dissociation between the language networks and the multiple-demand network (Blank et al. 2014). Although we examined the patterns of FC network dynamics to differentiate the functional specialization of each FC network during language processing, the results of the current study align with prior ROI studies demonstrating a functional dissociation between language-specific regions and language-related regions.

It is clinically important to identify the types of information each network is processing. For instance, surgical resection of the epileptogenic zone may be necessary for patients with drug-resistant focal epilepsy (Guery and Rheims 2021; Panigrahi and Jayalakshmi 2008; Ryvlin and Rheims 2008). Presurgical evaluation with multimodal brain imaging tools identifies the eloquent cortex, such as language-specific networks and language-related cognitive networks to avoid post-surgery impairments (Panigrahi and Jayalakshmi 2008; Ryvlin and Rheims 2008). Differentiating these networks should improve the accuracy of the epilepsy presurgical evaluation. Such information also will improve the prediction accuracy of language functions and recovery, which are heterogenous and challenging to predict, among patients with language disorders which are frequent after central nervous system (CNS) injury, including stroke (Cummings 2023; Kristinsson et al. 2022; Sinanović et al. 2011). (Billot et al. 2022; Charidimou et al. 2014; Watila and Balarabe 2015). Additionally, examining patterns of FC network dynamics without cognitive localizer tasks to differentiate network functional specialization may reduce the burden on those patients.

In considering our findings, several caveats should be considered. First, an alternative explanation may be that the observed networks emerged by chance. However, we think this is unlikely, as multiple controls were implemented to minimize this possibility, including statistical correction for multiple comparisons using Bonferroni correction, including the common networks that were statistically significant in at least 85% of all the participants in our analyses, in order to ensure that the networks analyzed were robust and functionally meaningful. Although we understand that additional networks may be activated as a function of individual variances, our results are restricted to the common networks found in a large majority of the 172 participants to ensure the robustness of our findings. Second, we conducted retrospective analyses, and behavioral metrics were not available. We have retrospectively interpreted that network dynamics that differ from the resting-state may be related to language processing, given that the subjects were actively engaged in performing such a task. However, we think that future studies should be prospectively designed to more conclusively determine the specific nature of the linguistic processing elements that are dynamically being processed in the non-default mode networks.

## Conclusion

The results of our study suggest that the temporal dynamics of neural networks are notably distinct in rs-fMRI and tb-fMRI, unlike spatial ROIs where the same functional network activation has been reported in both rs-fMRI and tb-fMRI. Our findings suggest that some aspects of fine-grain temporal patterns may indeed be reflected in dynamic functional connectivity BOLD signal correlation, and that dynamic patterns may be used to evaluate the types of information that networks are processing. Importantly, the results of the current study suggest that dynamic FC analyses of tb-fMRI may elucidate how language-specific networks perform differently from language-related networks and generalized cognitive networks in real time. Using such information may help to better understand a patient’s complete neurolinguistic profile at least with respect to language-related cognitive subnetworks vs. more language-specific subnetworks and may be exploited to develop improved neurobehavioral speech neurorehabilitation strategies following CNS injury.

## Supplementary Information

Supplementary Material 1

## Data Availability

This study used publicly available pre-existing datasets. Functional MRI data were obtained from the S1200 dataset from the Human Connectome Project, WU-Minn: [https://www.humanconnectome.org](https:/www.humanconnectome.org) . Additionally, de-identified data derived from the current study will be deposited in the Open Science Framework (OSF).

## Declarations

### Disclaimer

This research was supported by the Intramural Research Program of the National Institutes of Health (NIH). The contributions of the NIH author(s) are considered Works of the United States Government. The findings and conclusions presented in this paper are those of the author(s) and do not necessarily reflect the views of the NIH or the U.S. Department of Health and Human Services.
