# Supplementary material for: Differentiating language-specific from language-related network dynamics using dynamic functional connectivity analysis
Source: Cogn Neurodyn. 2026 Nov 10;20(1):217. doi: 10.1007/s11571-026-10579-0 (PMC13751807; doi:10.1007/s11571-026-10579-0)
Supplement: Supplementary file 1 [file 11571_2026_10579_MOESM1_ESM.docx]

**SUPPLEMENTAL MATERIAL**

**FIGURES**

**Figure S1. The pattern differences on mean Linear trend over time in network connections in tb-fMRI.**

A. **Mean linear trend over time in all left hemispheric network connections averaged across subjects.**
B. **Mean linear trend over time in all right hemispheric network connections averaged across subjects.**
C. **Mean linear trend over time in all inter-hemispheric network connections averaged across subjects.**

**Figure S2. Time-resolved dynamic functional connectivity trajectories for resting-state fMRI: the shaded parts indicate 95% confidence interval for each hemisphere.**

**Figure S3. Time-resolved dynamic functional connectivity trajectories for task-based fMRI: the shaded parts indicate 95% confidence interval for each hemisphere.**

**TABLES**

**Table S1. Desikan–Killiany atlas parcels and ROI–ROI edges contributing to the dynamic functional connectivity analyses for resting-state fMRI.**

NOTE: SigConn: significant connections

**Table S2. Desikan–Killiany atlas parcels and ROI–ROI edges contributing to the dynamic functional connectivity analyses for task-based fMRI.**

NOTE: SigConn: significant connections

A. **MEAN LINEAR TREND OVER TIME IN ALL LEFT HEMISPHERIC NETWORK CONNECTIONS AVERAGED ACROSS SUBJECTS.**

B. **MEAN LINEAR TREND OVER TIME IN ALL RIGHT HEMISPHERIC NETWORK CONNECTIONS AVERAGED ACROSS SUBJECTS.**

C. **MEAN LINEAR TREND OVER TIME IN ALL INTER-HEMISPHERIC NETWORK CONNECTIONS AVERAGED ACROSS SUBJECTS.**

| **ROI full REST** | **Hemisphere** | **ROI label** |  | **Edge category REST** | **N edges total** |
| --- | --- | --- | --- | --- | --- |
| lh-bankssts | LH | bankssts |  | sigConn LH | 3440 |
| lh-caudalmiddlefrontal | LH | caudalmiddlefrontal |  | sigConn RH | 3440 |
| lh-cuneus | LH | cuneus |  | sigConn inter | 6020 |
| lh-inferiorparietal | LH | inferiorparietal |  |  |  |
| lh-lateraloccipital | LH | lateraloccipital |  |  |  |
| lh-lingual | LH | lingual |  |  |  |
| lh-pericalcarine | LH | pericalcarine |  |  |  |
| lh-postcentral | LH | postcentral |  |  |  |
| lh-precentral | LH | precentral |  |  |  |
| lh-precuneus | LH | precuneus |  |  |  |
| lh-rostralmiddlefrontal | LH | rostralmiddlefrontal |  |  |  |
| lh-superiorfrontal | LH | superiorfrontal |  |  |  |
| lh-superiorparietal | LH | superiorparietal |  |  |  |
| lh-superiortemporal | LH | superiortemporal |  |  |  |
| lh-supramarginal | LH | supramarginal |  |  |  |
| lh-transversetemporal | LH | transversetemporal |  |  |  |
| rh-bankssts | RH | bankssts |  |  |  |
| rh-caudalmiddlefrontal | RH | caudalmiddlefrontal |  |  |  |
| rh-cuneus | RH | cuneus |  |  |  |
| rh-inferiorparietal | RH | inferiorparietal |  |  |  |
| rh-lateraloccipital | RH | lateraloccipital |  |  |  |
| rh-lingual | RH | lingual |  |  |  |
| rh-pericalcarine | RH | pericalcarine |  |  |  |
| rh-postcentral | RH | postcentral |  |  |  |
| rh-precentral | RH | precentral |  |  |  |
| rh-precuneus | RH | precuneus |  |  |  |
| rh-rostralmiddlefrontal | RH | rostralmiddlefrontal |  |  |  |
| rh-superiorfrontal | RH | superiorfrontal |  |  |  |
| rh-superiorparietal | RH | superiorparietal |  |  |  |
| rh-superiortemporal | RH | superiortemporal |  |  |  |
| rh-supramarginal | RH | supramarginal |  |  |  |
| rh-transversetemporal | RH | transversetemporal |  |  |  |

| **ROI full TASK** | **Hemisphere** | **ROI label** |  | **Edge category TASK** | **N edges total** |
| --- | --- | --- | --- | --- | --- |
| lh-bankssts | LH | bankssts |  | sigConn LH | 3440 |
| lh-caudalmiddlefrontal | LH | caudalmiddlefrontal |  | sigConn RH | 3440 |
| lh-cuneus | LH | cuneus |  | sigConn inter | 6020 |
| lh-inferiorparietal | LH | inferiorparietal |  |  |  |
| lh-lateraloccipital | LH | lateraloccipital |  |  |  |
| lh-lingual | LH | lingual |  |  |  |
| lh-pericalcarine | LH | pericalcarine |  |  |  |
| lh-postcentral | LH | postcentral |  |  |  |
| lh-precentral | LH | precentral |  |  |  |
| lh-precuneus | LH | precuneus |  |  |  |
| lh-rostralmiddlefrontal | LH | rostralmiddlefrontal |  |  |  |
| lh-superiorfrontal | LH | superiorfrontal |  |  |  |
| lh-superiorparietal | LH | superiorparietal |  |  |  |
| lh-superiortemporal | LH | superiortemporal |  |  |  |
| lh-supramarginal | LH | supramarginal |  |  |  |
| lh-transversetemporal | LH | transversetemporal |  |  |  |
| rh-bankssts | RH | bankssts |  |  |  |
| rh-caudalmiddlefrontal | RH | caudalmiddlefrontal |  |  |  |
| rh-cuneus | RH | cuneus |  |  |  |
| rh-inferiorparietal | RH | inferiorparietal |  |  |  |
| rh-lateraloccipital | RH | lateraloccipital |  |  |  |
| rh-lingual | RH | lingual |  |  |  |
| rh-pericalcarine | RH | pericalcarine |  |  |  |
| rh-postcentral | RH | postcentral |  |  |  |
| rh-precentral | RH | precentral |  |  |  |
| rh-precuneus | RH | precuneus |  |  |  |
| rh-rostralmiddlefrontal | RH | rostralmiddlefrontal |  |  |  |
| rh-superiorfrontal | RH | superiorfrontal |  |  |  |
| rh-superiorparietal | RH | superiorparietal |  |  |  |
| rh-superiortemporal | RH | superiortemporal |  |  |  |
| rh-supramarginal | RH | supramarginal |  |  |  |
| rh-transversetemporal | RH | transversetemporal |  |  |  |
